# Medical Photography in Dermatology: Quality and Safety in the Referral Process to Secondary Healthcare

**DOI:** 10.3390/diagnostics15121518

**Published:** 2025-06-14

**Authors:** Eduarda Castro Almeida, João Rocha-Neves, Ana Filipa Pedrosa, José Paulo Andrade

**Affiliations:** 1 Faculty of Medicine of University of Porto, Alameda Prof Hernâni Monteiro, 4200-319 Porto, Portugal; 2 CINTESIS@RISE, Biomedicine Department—Unity of Anatomy, Faculty of Medicine of University of Porto, Alameda Prof Hernâni Monteiro, 4200-450 Porto, Portugal; 3 Department of Dermatology and Venereology, ULS São João EPE, 4200-319 Porto, Portugal

**Keywords:** clinical photography, family medicine, referral pathway, triage, data security awareness, dermatologist

## Abstract

**Background:** Medical photography is widely used in dermatology referrals to secondary healthcare, yet concerns exist regarding image quality and data security. This study aimed to evaluate the quality of clinical photographs used in dermatology referrals, to identify discrepancies between specialties’ perceptions, and to determine the general awareness of proper storage and security of clinical photographs. **Methods**: A 43-question survey, based on previously validated questionnaires, was administered to general and family medicine (GFM) doctors and to dermatologists at an academic referral hospital in Porto, Portugal. The survey assessed demographics, photo-taking habits, perceived photo quality, adequacy of clinical information, and opinions on the role of photography in the referral process. Quantitative statistical methods were used to analyze questionnaire responses. **Results**: A total of 65 physicians participated (18 dermatologists and 47 GFM doctors). Significant differences were observed between the two groups. While 36.2% of GFMs rated their submitted photos as high- or very-high-quality, none of the dermatologists rated the received photos as high-quality, with 83.3% rating them as average (*p* = 0.012). Regarding clinical information, 46.8% of GFMs reported consistently sending enough information, while no dermatologists reported always receiving sufficient information (*p* < 0.001). Most respondents (76.9%) agreed that the quality of photographs is important in diagnosis and treatment. **Conclusions**: The findings reveal a discrepancy between GFM doctors’ and dermatologists’ perceptions of photograph quality and information sufficiency in dermatology referrals. Standardized guidelines and educational interventions are necessary to improve the quality and consistency of clinical photographs, thereby enhancing communication between healthcare providers and ensuring patient data privacy and security.

## 1. Introduction

The recording of medical photographs is an essential component across various medical specialties and is increasingly common today due to easier access to mobile devices [1]. The significance of photography has been well-documented in various medical and surgical fields, including plastic surgery, ophthalmology, and dermatology. In these areas, photography supports diagnostic processes, disease monitoring, tracking treatment responses, surveillance of high-risk patients, and obtaining second opinions. It is also valuable for academic purposes, including education, conference presentations, and publication in scientific journals [2,3,4].

In Portugal, a 2022 study found that 93% of participating physicians in an academic referral hospital across various specialties utilized medical photography in their clinical practice. Most clinical photographs were taken using smartphones [5,6]. Despite its frequent use, obtaining an accurate clinical photograph can be challenging without proper training [7,8]. As a result, image quality is not always optimal for accurate clinical assessment. Poor framing, inadequate lighting, or blurred images can hinder effective diagnosis or evaluation [9]. The availability of clear and detailed photographs improves the triage process. Dermatologists can determine the urgency of a referral more accurately, ensuring that patients with severe or urgent conditions receive timely care, while less critical cases are managed appropriately [9]. This highlights the need for effective information dissemination and the standardization of medical photography practices to facilitate communication among healthcare providers [4].

In addition to the technical aspects of photography, patient data privacy and security are critical concerns, as health information is considered among the most sensitive and personal information individuals possess [10]. Patients generally perceive medical photography positively when it is used for diagnosis and treatment [11]. Studies have reported that most patients believe photography enhances their quality of care by facilitating better communication between primary care providers and specialists [12]. However, concerns about privacy and consent must be addressed to maintain trust and ensure compliance with ethical standards [11,13]. The use of smartphones, particularly in hospital and clinical settings, demands attention to secure storage, image transfer, and medico-legal considerations [14]. Studies from other countries indicate that 70% to 92% of dermatologists seek verbal consent for clinical photographs. Yet, only 15% of dermatologists document this in the clinical record, and a mere 17% obtain written consent [15,16].

In Portugal, general and family medicine (GFM) is a recognized specialty. These physicians are responsible for the initial assessment and management of patients in primary care settings as well as for referring patients to hospital-based specialties when necessary. Particularly, they refer patients to the hospital’s dermatology specialty. The referring physician should send relevant clinical information and attach a photograph of the lesion or nevus, which will be the reason for consultation. Often, these photographs are not recorded with adequate quality to allow for a correct pre-assessment of patients by dermatology, which may hinder the planning and management of the best possible plan for the patient. This process holds even greater importance in the case of urgent dermatology referrals for the evaluation of a lesion concerning skin cancer, which should be triaged and processed with appropriate urgency [17].

The primary objective of this study is to provide valuable context to the existing literature on medical photography, highlighting current practices and areas for improvement in the Portuguese healthcare system, particularly in the referral pathway. Specifically, the objective is to understand how the recorded photographs can impact and influence the referral process, whether in establishing patient priority or not, and how to optimize this process. A secondary objective of this study was to raise awareness about the proper storage and security of photographs to ensure patient privacy. Ultimately, we aim to encourage standardization in photograph acquisition through the development of an informative guide, while also promoting digital security literacy, a growingly relevant issue.

## 2. Materials and Methods

A 43-question questionnaire was developed, based on the precedent set by previous studies [5,15,18].

The target population for this study consisted of two specialties: GFM doctors and dermatologists, from ULS São João, an academic tertiary referral center located in Porto, Portugal. Inclusion criteria encompassed practicing GFMs and dermatologists with at least one year of clinical experience.

A pilot study was initially performed with 20 participants to evaluate the feasibility and clarity of the survey. Based on their feedback, adjustments were made to the questionnaire to improve its comprehensibility and relevance.

The questionnaire consisted of three main sections. Demography questions (Q) (Q1–Q6) gathered information from all participants on gender, age group, years of practice, medical career status, specialty, and smartphone usage for clinical practice. The second part was divided into two subsections: one for dermatologists (Q7–Q13) and another for GFMs (Q14–Q20). Dermatologists evaluated the frequency and quality of photographs received in referrals, the adequacy of clinical information, and the importance of photo quality in diagnosis and treatment. GFM doctors assessed their practices when sending referrals, including photo-taking habits, perceived quality of photos sent, and the importance of photography in the referral process. For example, while Q8 asked dermatologists to evaluate the quality of received photos, Q15 asked GFMs to rate the quality of the images they submit.

The third part (Q21–Q43) was a general habits questionnaire answered by all participants, regardless of specialty. This section explored various aspects of clinical photography practices, consent practices, data security and storage methods, photo transmission practices, and training needs related to clinical photography and data security.

This structure enabled a comprehensive assessment of clinical photography practices in dermatology referrals, allowing direct comparison between the perspectives of those sending referrals and those receiving them, while also providing insights into general habits and practices across both specialties.

The survey consisted of a mix of multiple-choice, Likert-scale questions without logic-based branching as well as open-ended questions. A copy of the whole questionnaire in English is included in Supplementary Material S1. The open-ended questions (Q13, Q20, and Q43) were not mandatory and were only included to allow participants to share additional thoughts, insights, or comments on the topic. All other questions were required for submission to ensure data completeness and quality. Data collected in some questions are not discussed in this paper, as they are not within the objectives of the present study.

Answers to our questionnaire were collected in November 2024, and two different approaches were used for data collection. The survey was presented to the dermatologists in person during a departmental meeting, and a link to fill out the survey was shared. To the GFM doctors, the survey was shared via email with all 29 local coordinators of each primary care health unit (USF) that belongs to the referral area of the ULS S. João, and then shared among the doctors working there.

Participation in the survey was entirely voluntary. Participants provided informed consent through a mandatory checkbox before beginning the study. The title page included detailed information about participants’ rights, including their right to withdraw their data at any time. No identifiable data were collected, ensuring anonymity. The study protocol was approved by the local Ethics Committee (protocol 364-2024). Participants’ informed consent was handled accordingly, and all data processing was anonymous and in accordance with the European Union General Data Protection Regulation (GDPR) [19]. This study adheres to the Checklist for Reporting Results of Internet E-Surveys (CHERRIES) criteria [20].

The survey was hosted on Microsoft Forms® (Microsoft Corporation, Redmond, WA, USA). All responses were anonymous, with no personal identifiers collected. Data were securely stored by the corresponding author, ensuring compliance with GDPR. Data will be retained for academic purposes for a period not exceeding two years.

Descriptive statistics of the most relevant questions for the aims of this paper are presented as absolute counts and proportions. Since several questions allowed participants to select more than one answer, each answer possible was treated as a dichotomous variable (yes/no). Questions were analyzed by specialty to explore potential differences between groups. Data analyses were conducted using IBM SPSS Statistics (IBM Corp., release 2023. IBM SPSS Statistics for Windows, version 29.0.2.0, Armonk, NY, USA). Data visualization and graphical representations were created using Microsoft Excel (Office 365, version 2022, Microsoft Corporation, Redmond, WA, USA). Missing data were avoided as all questions were mandatory for submission. The pilot study results were used solely to refine the questionnaire and were excluded from the final analysis.

Normally distributed data were presented as mean and standard deviation (SD), while categorical variables were expressed as frequency and corresponding percentage. For univariable analysis, χ2 or Fisher’s test was used for qualitative data, and Student’s T-test for quantitative data. A statistical significance threshold of less than 0.05 was considered.

## 3. Results

A total of 65 physicians participated in the survey, including 18 dermatologists, which corresponds to approximately 82% of the 22 dermatologists working at ULS S. João, and 47 GFM doctors working in the primary care of ULS S. João, which corresponds to approximately 18% of the total 259.

More than half of the participants were female (72.3%) and residents (76.9%). Most of the sample (60.0%) were 40 years old or younger, and only four doctors (6.2%) had a smartphone exclusively for work practice. No significant differences existed between specialties in gender, age, or years of practice. The table referring to this section can be consulted in Supplementary Material S2.

A comparison of the perspectives between specialties, specifically regarding the clinical photos involved in the referral process, is shown in Table 1. When evaluating photo quality (Q8 and Q15), 36.2% of GFMs rated their submitted photos as high- or very-high-quality, whereas no dermatologists rated their submitted photos as high-quality, with 83.3% rating them as average (*p* = 0.012). Regarding clinical information (Q10 and Q17), 46.8% of GFM doctors claimed that they always send enough information in 100% of orders. In contrast, no dermatologists reported receiving enough clinical information. Most dermatologists (66.7%) indicated receiving enough clinical information in 50–75% of referrals (*p* < 0.001).

**Figure 1:**
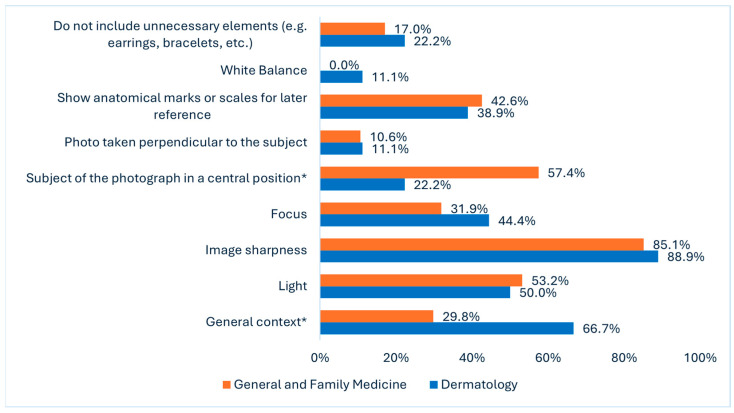
Replies from two distinct questions: Q9 (dermatologists) “About the following characteristics, which do you consider not to be correctly met, in general, in the photographs received?”; Q16 (GFM) Regarding the following characteristics, which ones do you consider when taking a photograph to be sent specifically in the dermatological referral?” (* *p* = 0.01).

Figure 1 presents data from two distinct questions: Q9 (dermatology) and Q16 (GFM). Of GFM doctors, 85.1% claimed to consider image sharpness, and 88.9% of dermatologists considered that this feature was not correctly met. Light (53.2% vs. 50.0%) and the presence of anatomical marks or scales for later reference (42.6% vs. 38.9%) had similar responses. Statistically significant differences were found in two categories for the subject of the photograph in a central position. In this question, 57.4% of GFM doctors reported considering this aspect, and only 22.2% of dermatologists said it was not considered (*p* = 0.01). For general context, i.e., including enough surrounding anatomical area to provide orientation and location, 29.8% of GFM doctors replied that they consider it, whereas 66.7% of dermatologists consider this feature missing (*p* = 0.01). On white balance, we found that no GFM doctors (0.0%) considered this feature, and only 11.1% of dermatologists reported missing it.

Regarding the assessment of opinions from both specialties on the role of clinical photography in the referral process, 76.9% of all respondents (70.2% of GFM physicians and 94.4% of dermatologists, *p* = 0.215) believe that photograph quality is important in the diagnosis and treatment of patients. Additionally, 83.1% of all respondents (78.7% of GFM doctors and 94.4% of dermatologists, *p* = 0.115) believe that improvements in the referral process, such as enhancing the quality of clinical photographs and the information provided, could have a significant positive impact on patient care and clinical outcomes.

Key differences in clinical photography practices between GFM doctors and dermatologists are highlighted in Table 2. The full table results for this section of the questionnaire can be consulted in Supplementary Material S2. On the frequency of clinical photography use (Q21), dermatologists reported taking clinical photos significantly more often than GFM doctors, with 55.6% of dermatologists taking photos several times a day compared to only 2.1% of GFM doctors (*p* < 0.001). Regarding the devices used (Q22), most practitioners of both groups (90.8%) used personal smartphones for clinical photography. However, dermatologists were significantly more likely to use specialized equipment such as dermatoscopes (44.4% vs. 0%, *p* < 0.001).

**Figure 2:**
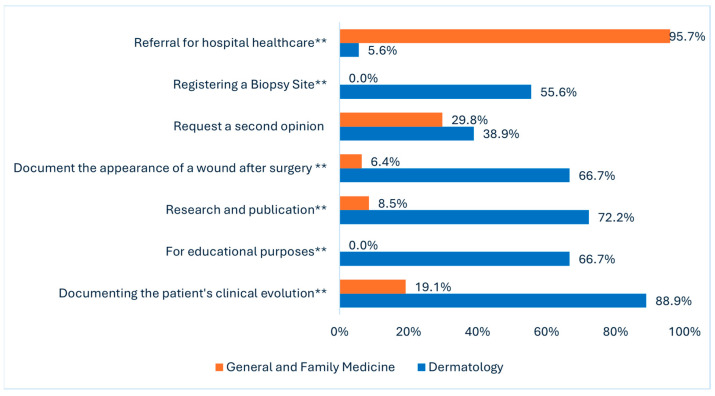
Replies to Q23: “What are the main reasons for photographic registration?” (** *p* < 0.001).

The primary reasons for taking clinical photographs (Q23) differed significantly between the two groups. Dermatologists primarily used photography for documenting clinical evolution (88.9%, *p* < 0.001), research purposes (72.2%, *p* < 0.001), educational purposes (66.7%, *p* < 0.001), registering a biopsy site (55.6%, *p* < 0.001), and documenting the appearance of a wound (66.7%, *p* < 0.001). GFM doctors primarily used it for referrals to hospital healthcare (95.7%, *p* < 0.001) (Figure 2). Both reported using photography to request a second opinion (29.8% vs. 38.9%, *p* = 0.558).

Regarding photographic characteristics (Q24), dermatologists are more likely to consider the general context (83.3% vs. 42.6%, *p* = 0.005), focus (72.2% vs. 36.2%, *p* = 0.013), and white balance (6.2% vs. 0.0%, *p* = 0.005) when taking photos. Training in clinical photography (Q26) is rare overall, but it is significantly more common among dermatologists, with 22.2% reporting prior training, compared to none among GFMs (*p* = 0.005).

Consent practices (Q27 and Q28) vary, with 95.7% of GFM doctors always obtaining consent compared to 77.8% of dermatologists (*p* = 0.015). Most participants request consent verbally (84.6%), with GFM reporting more routinely to record the consent in the clinical diary (38.3% vs. 5.6%, *p* = 0.013). Photo storage practices (Q29) reveal further differences: 94.4% of dermatologists store photos on personal devices compared to only 23.4% of GFM doctors (*p* < 0.001).

Dermatologists reported higher usage of security measures (Q31) compared to GFMs. Access codes were used by 50% of dermatologists, and 61.1% used automatic phone locks. In contrast, 17% and 19.1% of GFMs used them, respectively (*p* = 0.011 and *p* = 0.002). For transmitting clinical photos (Q32), GFMs favored email (63.8%) and applications designed for clinical practice (31.9% vs. 0.0%, *p* = 0.006), while dermatologists used WhatsApp or Facebook Messenger more frequently (44.4% vs. 10.6%, *p* = 0.005).

Regarding photo quality (Q34), 50% of dermatologists rated their submitted photos as high- or very-high-quality, compared to 38.3% of GFMs, though this difference was not statistically significant (*p* = 0.292). Most respondents (81.6%) considered sending and receiving clinical photos to be important or very important for effective patient management (Q35), and the perceived importance did not significantly differ between specialties (*p* = 0.180).

Most respondents (95.4%) believe they provide appropriate contextualization for clinical cases when sending photographs (Q36), either always or most of the time, and there is a statistically significant difference between specialties (*p* = 0.042). Dermatologists reported receiving clinical photos (Q37) more frequently, with 38.9% receiving them daily compared to only 2.1% of GFMs (*p* < 0.001).

Most participants (83.1%) reported no clear workplace guidelines on a formal procedure for adding clinical photos from the smartphone to a patient’s record (Q39). And 90.8% also reported no clear workplace guidelines on how to take an accurate medical photo using a smartphone (Q40). Both groups express interest in receiving training, with 55.4% wanting guidance on taking good medical photos (Q41) and 66.2% seeking training on security, transmission, and storage practices (Q42).

In our open-ended questions to assess physicians’ thoughts on the subject, we obtained eight answers. GFMs highlighted their main concerns about the necessity of using their personal mobile devices for capturing and transmitting images, due to the lack of a device provided by their work unit, which raises ethical, legal, and logistical issues [13]. Some argue they should not be responsible for photographing and handling patient images, as this task falls outside their core medical duties and introduces additional administrative burdens and workload.

## 4. Discussion

Substantial discrepancies were identified between GFM physicians and dermatologists in their perceptions and practices concerning clinical photography within the referral pathway, underscoring the prevailing challenges in this domain. Notably, a marked divergence emerged in the assessment of photographic quality: whereas 36.2% of GFM physicians rated the images they submitted as high- or very-high-quality, none of the dermatologists evaluated the received photographs as high-quality, with 83.3% classifying them as average (Table 1). More specifically, the areas in photo quality that fall short of dermatologists’ expectations include image sharpness, lighting, and the inclusion of anatomical marks or scales for reference, which are important features of medical photography [21].

Another notable discrepancy emerged regarding the perceived adequacy of clinical information included in referrals. Whereas 46.8% of GFM physicians reported that they consistently provided sufficient clinical details, none of the dermatologists indicated that they always received adequate information. This communication gap could potentially lead to delays in diagnosis and treatment, emphasizing the need to clarify the information necessary and expected to be sent when referring a patient [8,22].

A qualitative study conducted in Singapore exploring the experiences of family doctors and dermatologists with teledermatology in primary care settings corroborates some of the identified challenges. Specifically, this study highlighted the need to streamline administrative referral processes to reduce their burden and disruption to clinical workflows. Furthermore, dermatologists in that study emphasized that the quality of photographic images directly impacts the accuracy and reliability of their diagnoses and treatment recommendations, which aligns with our findings [23].

Most respondents (76.9%) believed that the quality of photographs plays an important role in diagnosis and treatment, with an even higher percentage among dermatologists (94.4%). The quality of the photographs is also emphasized by most papers related to dermatologic images [24]. Without correct exposure and sharpness, the value of a clinical photograph declines [24].

On the general habits question, it was found that dermatologists primarily use clinical photography for documenting clinical evolution (88.9%), research purposes (72.2%), and educational purposes (66.7%), which is consistent with other studies [3,15]. In contrast, GFMs primarily used clinical photography to refer patients to hospital healthcare (95.7%). Interestingly, both groups reported similar rates of using photography to request a second opinion. This shared practice aligns with the growing trend of utilizing clinical photography for telemedicine and remote consultations, as noted in previous studies [25,26].

Regarding technical aspects, white balance is a fundamental parameter in medical imaging that directly influences diagnostic accuracy by ensuring consistent color representation across photographic documentation [21]. Improper calibration introduces artificial hue shifts that compromise quantitative assessments of melanin distribution, vascular lesions, and inflammatory markers—particularly in patients with darker skin tones where melanin density obscures erythema detection [27,28]. Our results showed that no GFMs consider white balance when taking a photo, and only 6.2% of dermatologists consider it. This indicates the need for training on such an essential matter [21].

**Figure 3:**
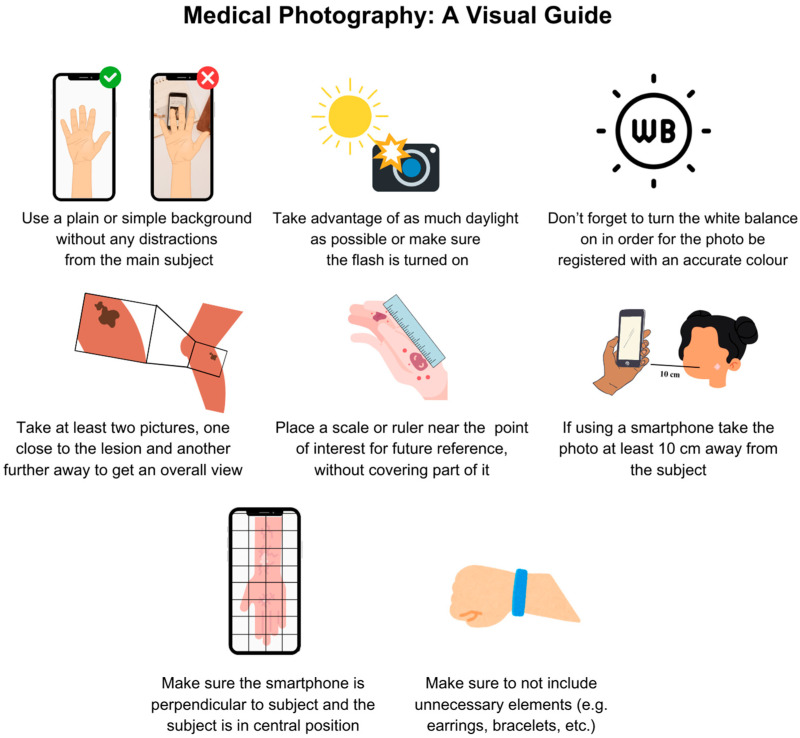
Key recommendations for capturing accurate and useful medical photographs using a smartphone or similar device.

Capturing an accurate medical photograph can be a challenging task without appropriate training or equipment, which, in turn, impacts the quality of the photo [9]. Recognizing this gap and the interest shown in training by physicians, we developed a concise flyer with basic information based on a comprehensive review of the existing literature and best practices on how to take an accurate medical photograph [3,8,9,24,29,30]. This user-friendly guide addresses common gaps in clinical photography. It provides essential tips for producing high-quality images suitable for dermatological assessment, which can be used for both physicians and patients (Figure 3).

Furthermore, investment in equipment for GFM doctors should be considered. We found a significant gap in the use of more specific equipment, with 44.4% of dermatologists using dermatoscopes compared to no use of these specialized devices by GFMs.

It was found that 90.8% of respondents used personal smartphones for clinical photography, as seen in previous studies [5,18,31], which raises concerns regarding data privacy [2]. Healthcare institutions must implement robust guidelines for secure image capture, transmission, and storage and provide adequate training and information to their healthcare professionals. Most physicians claimed not to be aware of its existence (83.1%), a value that is very similar to that of another study [6]. The mobile device should be protected using a strong passcode, and any cloud-based backup systems should be disabled before use [4,5]. A dedicated healthcare mobile device capture application with secure encryption is recommended [9,19]. In our study, dermatologists reported higher usage of security, using more access codes than GFMs. On the other hand, GFM doctors reported greater usage of applications designed for clinical practice, primarily due to the referral process and email. Dermatologists reported using WhatsApp or Facebook Messenger more frequently, a trend also observed in other studies [5,18,32]. However, this practice does not meet GDPR or United States Health Insurance Portability and Accountability Act (HIPAA) compliance, as discussed elsewhere [2,19,33].

Most participants request verbal consent (83%), but only 16% of practitioners systematically document this in the patient diary. These results closely align with those reported in previous studies [5,6,15,18], underscoring a persistent pattern in consent practices across various healthcare settings. This concordance not only validates these findings but also highlights an ongoing challenge in the systematic documentation of patient consent, suggesting a need for improved protocols and increased awareness in this critical aspect of patient care [3,26,34].

To mitigate ongoing challenges and utilize medical photography more efficiently, some studies provide recommendations for safe clinical photography, along with practical guidelines [2,4]. First, it is essential to obtain the patient’s informed consent for photography, covering the purpose of clinical photos, access control, identity protection, and image storage [13]. After image capture, images should be securely saved directly to the patient’s electronic record from the device that captured the image [35]. Lastly, it is important to establish image file exchange standards to promote interoperability. This is essential for both provider-to-provider communication and image sharing with patients [4]. Implementing transmission standards ensures data transmission between trusted and verified senders and receivers for both providers and patients [4].

By implementing measures, healthcare organizations can mitigate risks of data breaches, ensure GDPR compliance and HIPAAA compliance, and improve the overall quality and security of clinical photography in dermatology referrals [4]. Given the prevalence of smartphone use, there may be opportunities to develop specialized apps or tools to improve photo quality and standardization and relieve the workload while making the referral process more efficient [34]. A study conducted in the US concluded that point-of-care medical photography using a secure mobile, electronic health record-integrated application can become a new standard of care for clinical documentation and may facilitate continuity across the continuum of care with multiple providers who see a patient [36].

The relatively low response rate from GFMs (18%) compared to dermatologists (82%) may limit the generalizability of findings and may introduce selection bias. The single-center design further limits generalizability to other Portuguese regions with distinct resource profiles. It has also limited external validity in other countries’ practices, which utilize different referral systems.

## 5. Conclusions

Achieving consistency in clinical photography remains a challenge due to variability in equipment, techniques, and patient characteristics [7,8]. High-quality images enable accurate diagnoses, improve triage efficiency, reduce waiting times, and minimize unnecessary consultations [4,34,37]. It is essential to recognize the challenges physicians encounter during this process, as their role as active participants can be significantly enhanced when equipped with appropriate measures and support, enabling them to make substantial health-related changes that are crucial for optimizing patient care and clinical outcomes. These challenges can be addressed through standardized guidelines and educational initiatives [2,4]. The implementation of solid guidelines could not only improve communication between physicians and specialists but also enhance patient and medical literacy regarding safe and effective methods for capturing and sharing photographs [15].

Future research could explore innovative solutions, such as mobile apps or other tools, to enhance photographic quality in the referral process, ultimately leading to improved patient care and more efficient use of healthcare resources.

## Figures and Tables

**Table 1:** Comparative analysis of perspectives.

	Dermatology	GFM	*p*-Value
	Q7 How often **does a referred patient** from the specialty of general and family medicine have a photograph attached to the file?	Q14 How often **do you attach** a clinical photograph when referring a patient to dermatology hospital healthcare?	
Mandatory	7 (38.9%)	34 (72.3%)	**<0.001**
Always	1 (5.6%)	12 (25.5%)	
Often	10 (55.6%)	1 (2.1%)	
Sometimes or never	0 (0.0%)	0 (0.0%)	
	Q8 How do you evaluate the quality of the photos **received** in general?	Q15 How would you rate the quality of the photos you **submit** in general?	
Low	3 (16.7%)	6 (12.8%)	**0.012**
Average	15 (83.3%)	24 (51.1%)	
High or Very High	0 (0.0%)	17 (36.2%)	
	Q10 How often is enough clinical information **sent to you?**	Q17 How often **do you send** enough clinical information?	
100% of orders	0 (0.0%)	22 (46.8%)	**<0.001**
75–99% of orders	3 (16.7%)	23 (48.9%)	
50–75% of orders	12 (66.7%)	1 (2.1%)	
<50% of orders	3 (16.7%)	1 (2.1%)	

**Table 2:** Comprehensive analysis of habits and behavioral patterns.

Questions	Total	GFM	Dermatology	*p*-Value
Q21 How often do you take pictures of patients?				
Several times a day	11 (16.9%)	1(2.1%)	10 (55.6%)	**<0.001**
Daily	5 (7.7%)	2 (4.3%)	3 (16.7%)	
Weekly	37 (56.9%)	32 (68.1%)	5 (27.8%)	
Monthly	11 (16.9%)	11 (23.4%)	0 (0.0%)	
Never	1 (1.5%)	1 (2.1%)	0 (0.0%)	
* Q28 How do you ask the patient for consent when taking a photo?				
Verbally	55 (84.6%)	37 (78.7%)	18 (100.0%)	0.051
Written	14 (21.5%)	12 (25.5%)	2 (11.1%)	0.316
Routine recording in the clinical diary	19 (29.2%)	18 (38.3%)	1 (5.6%)	**0.013**
Express consent	4 (6.2%)	4 (8.5%)	0 (0.0%)	0.569
Implied consent	3 (4.6%)	2 (4.3%)	1 (5.6%)	1.0
Patients themselves share images taken by them before the appointment	11 (16.9%)	10 (21.3%)	1 (5.6%)	0.265
* Q29 Where do you store patient photos?				
Personal device	28 (43.1%)	11 (23.4%)	17 (94.4%)	**<0.001**
Personal device for practice only	8 (12.3%)	4 (8.5%)	4 (22.2%)	0.202
Institutional device	24 (36.9%)	20 (42.6%)	4 (22.2%)	0.159
Clinical patient diary	19 (29.2%)	18 (38.3%)	1 (5.6%)	**0.013**
Specific institutional server	4 (6.2%)	3 (6.4%)	1 (5.6%)	1.0
Personal cloud (Google Drive, iCloud, Others)	1 (1.5%)	0 (0.0%)	1 (5.6%)	0.277
Institutional hard drive or equivalent	10 (15.4%)	10 (21.3%)	0 (0.0%)	0.051
Never store	2 (3.1%)	2 (4.3%)	0 (0.0%)	1.0
Not applicable	2 (3.1%)	2 (4.3%)	0 (0.0%)	1.0
* Q31 Do you use any of the following methods to protect your information?				
Access code	17 (26.2%)	8 (17.0%)	9 (50.0%)	**0.011**
Automatic phone lock	20 (30.8%)	9 (19.1%)	11 (61.1%)	**0.002**
Encryption	3 (4.6%)	1 (2.1%)	2 (11.1%)	0.183
Not applicable	35 (53.8%)	31 (66.0%)	4 (22.2%)	**0.002**
* Q32 What method do you use to send or receive clinical photos?				
Email	37 (56.9%)	30 (63.8%)	7 (38.9%)	0.095
WhatsApp or Facebook Messenger	13 (20.0%)	5 (10.6%)	8 (44.4%)	**0.005**
Application specifically designed for clinical practice.	15 (23.1%)	15 (31.9%)	0 (0.0%)	**0.006**
Not applicable	6 (9.2%)	1 (2.1%)	5 (27.8%)	**0.005**

See the complete table in Supplementary Material S2. Questions marked with * have multiple answers.

## Data Availability

The data that support the findings of this study are not openly available due to privacy and are available from the corresponding author upon reasonable request.

## Supplementary Materials

The following supporting information can be downloaded at https://www.mdpi.com/article/10.3390/diagnostics15121518/s1: Supplementary Material S1: Medical photography questionnaire: a translated copy of the distributed questionnaire; Supplementary Material S2: Full table of results.

## Abbreviations

The following abbreviations are used in this manuscript:

GFM	General and Family Medicine
ULS	Unidade Local de Saúde
GDPR	General Data Protection Regulation
Q	Question
HIPAA	Health Insurance Portability and Accountability Act
