# Supplementary material for: Medical Photography in Dermatology: Quality and Safety in the Referral Process to Secondary Healthcare
Source: Diagnostics (Basel). 2025 Jun 14;15(12):1518. doi: 10.3390/diagnostics15121518 (PMC12191595; doi:10.3390/diagnostics15121518)
Supplement: Supplementary file 1 [file diagnostics-15-01518-s001.zip › Supplementary Material S1 Questionnaire.pdf]

Supplementary Material S1

Questionnaire

Part 1- Demography

1. Gender

- Male
- Female
- Other

2. Age group

- 20-40 years
- 41-60 years
- 61 years of age or older

3. How many years have you been practicing in your specialty, including medical residency?

- < 5 years
- 5 – 10 years
- 11 – 20 years old
- More than 20 years

4. Do you have a smartphone exclusively for clinical practice at work?

- No
- Yes

5. Medical Career

- Specialist
- Resident

6. Specialty

- Dermatology
- General and Family Medicine

Part 2.1 – Dermatology Perspective

Considering the context of the photographs sent by General and Family Medicine doctors to Dermatology in **the process of referring** a patient to hospital care, answer the following questions:

7. How often does a referred patient from the specialty of general and family medicine have a photograph attached to the file?

- They can only reference if a photo is attached
- Always
- Often
- Sometimes
- Rarely
- Never

8. How do you evaluate the quality of the photos received in general?

- Low
- Average
- High
- Very high

9. About the following characteristics, which do you consider **not** to be correctly met, in general, in the photographs received? (fill in all that apply)

- Light
- General context
- Subject of the photograph in a central position
- Show anatomical marks or scales for later reference
- Photo taken perpendicular to the subject
- Focus

- Image sharpness
- True-to-life color
- White Balance
- Do not include unnecessary elements (e.g. earrings, bracelets, etc.)
- Other:________

10. How often is enough clinical information sent to you?

- 100% of orders
- 75 - 99% of orders
- 50 - 75% of orders
- 25 - 50% of orders
- < 25% of orders

11. Do you consider that the quality of the photographs received in the referral process from primary health care to hospital health care plays an important role in the diagnosis and treatment of patients?

- Yes, it can make a difference.
- Yes, but I don't think there will be a big impact.
- Neutral
- It does not play a major role.
- Other:_____________

12. Do you think that the referral process can be improved and have a relevant impact on improving health care?

- Yes
- No
- Neutral

13. Other comments on the subject: (non-obligatory open-ended question)

Part 2.2 – Perspective of General and Family Medicine

Considering the context of the photographs sent in the process of referring a patient to hospital care, answer the following questions:

14. How often do you attach a clinical photograph when referring a patient to dermatology hospital healthcare?

- I can only reference if I have a photo attached
- Always
- Often
- Sometimes
- Rarely
- Never

15. How would you rate the quality of the photos you submit in general?

- Low
- Average
- High
- Very high

16. Regarding the following characteristics, which ones do you consider when taking a photograph to be sent specifically in the dermatological referral? (fill in all that apply)

- Light
- General context
- Subject of the photograph in a central position
- Show anatomical marks or scales for later reference
- Photo taken perpendicular to the subject
- Focus
- Image sharpness
- True-to-life color
- White Balance

- ☐ Do not include unnecessary elements (e.g. earrings, bracelets, etc.)
- ☐ Other:________

17. How often do you send enough clinical information?

- ☐ 100% of orders
- ☐ 75 - 99% of orders
- ☐ 50 - 75% of orders
- ☐ 25 - 50% of orders
- ☐ < 25% of orders

18. Do you consider that the quality of the photographs sent in the referral process from primary health care to hospital health care plays an important role in the diagnosis and treatment of patients?

- ☐ Yes, it can make a difference.
- ☐ Yes, but I don't think there will be a big impact.
- ☐ Neutral
- ☐ It does not play a major role.
- ☐ Other:_____________

19. Do you think that the referral process can be improved and have a relevant impact on improving health care?

- ☐ Yes
- ☐ No
- ☐ Neutral

20. Other comments on the subject: (non-obligatory open-ended question)

Part 3 – General Habits

This part of the survey aims to study the recording habits and the quality and safety of photographs taken in a general context.

21. How often do you take pictures of patients?

- ☐ Several times a day
- ☐ Daily
- ☐ Weekly
- ☐ Monthly
- ☐ Annually
- ☐ Never

22. What device do you use to take pictures of patients?

- ☐ Personal smartphone
- ☐ Personal Digital Camera
- ☐ Smartphone used exclusively for clinical practice
- ☐ Exclusive digital camera for clinic
- ☐ Dermatoscope
- ☐ Not applicable
- ☐ Other:____________

23. What are the main reasons for photographic registration? (fill in all that apply)

- ☐ Documenting the patient's clinical evolution
- ☐ Request a second opinion
- ☐ For educational purposes
- ☐ Research and publication
- ☐ Registering a Biopsy Site
- ☐ Document the appearance of a wound after surgery
- ☐ Referral for hospital healthcare
- ☐ Not applicable
- ☐ Other:____________

24. When taking a photo, what characteristics do you consider? (fill in all that apply)

- ☐ Light
- ☐ General context
- ☐ Subject of the photograph in a central position
- ☐ Show anatomical marks or scales for later reference
- ☐ Photo taken perpendicular to the subject
- ☐ Focus
- ☐ Image sharpness
- ☐ True-to-life color
- ☐ White Balance
- ☐ Do not include unnecessary elements (e.g. earrings, bracelets, etc.)
- ☐ Not applicable
- ☐ Other:_________

25. Do you feel confident in your ability to take an accurate clinical photograph?

- ☐ Yes, I feel confident in my abilities.
- ☐ Yes, but there is still room for improvement.
- ☐ I feel partially confident
- ☐ I don't feel confident when I take a clinical photo.
- ☐ Not applicable

26. At some point in your career, did you have training in clinical photography?

- ☐ Yes
- ☐ No

27. How often do you ask for consent when taking a photo?

- ☐ I always ask
- ☐ I ask often
- ☐ When I remember
- ☐ Rarely
- ☐ Never
- ☐ Not applicable

28. How do you ask the patient for consent when taking a photo? (fill in all that apply)

- ☐ Verbally
- ☐ Written
- ☐ Routine recording in the clinical diary
- ☐ Express consent
- ☐ Implied consent
- ☐ Patients themselves share images taken by them before the appointment
- ☐ Not applicable

29. Where do you store patient photos? (fill in all that apply)

- ☐ Personal Device
- ☐ Personal device for business use only
- ☐ Institutional arrangement
- ☐ Clinical patient diary
- ☐ Specific institutional server
- ☐ Personal Cloud (Google Drive, iCloud, Others)
- ☐ Personal Cloud for Professional Use Only (Google Drive, iCloud, Others)
- ☐ Personal hard drive or equivalent
- ☐ Personal hard drive or equivalent for professional use only
- ☐ Institutional hard drive or equivalent
- ☐ Not applicable
- ☐ Other:___________

30. When do you delete photos from your device?

- ☐ Immediately after storing them elsewhere.
- ☐ When I remember after storing them somewhere other than the device.
- ☐ They always stay on the device.
- ☐ It does not apply.

31. Do you use any of the following methods to protect your information? You can choose more than one option:

- ☐ Access code
- ☐ Automatic phone lock
- ☐ Encryption
- ☐ Not applicable
- ☐ Other:_________

32. What method do you use to send or receive clinical photos? (fill in all that apply)

- ☐ Text message
- ☐ Email
- ☐ WhatsApp or Facebook Messenger
- ☐ Application specifically designed for clinical practice.
- ☐ Not applicable
- ☐ Other:_________

33. Who do you usually send clinical photographs to? (fill in all that apply)

- ☐ Dermatology
- ☐ General and Family Medicine
- ☐ Patient
- ☐ Other specialties
- ☐ Not applicable

34. How would you rate the quality of the photos you submit in general?

- ☐ Low
- ☐ Average
- ☐ Discharge
- ☐ Very high
- ☐ Not applicable

35. How important is it for you, as part of effective patient management, to be able to send and receive clinical photos?

- ☐ Very important
- ☐ Important
- ☐ Not that important
- ☐ Neutral
- ☐ Not particularly important
- ☐ Not relevant to my clinical practice

36. Do you consider that you usually contextualize the clinical case of the photograph sent in an appropriate way?

- ☐ Yes, always
- ☐ Yes, most of the time
- ☐ No
- ☐ Not sure
- ☐ Not applicable

37. How often do you receive a clinical photo by message or email?

- ☐ Daily
- ☐ Weekly
- ☐ Monthly
- ☐ Rarely
- ☐ I don't get paid

38. Who sends you clinical photos? (fill in all that apply)

- ☐ Dermatology
- ☐ General and Family Medicine
- ☐ Patient
- ☐ Other specialties
- ☐ Not applicable

39. Does your workplace have a formal procedure or method for adding clinical photos from your smartphone to a patient's record?

- ☐ Yes
- ☐ No
- ☐ I'm not sure.

40. Does your workplace provide clear guidelines on the use of smartphones for clinical photography?

- ☐ Yes
- ☐ No
- ☐ Not sure

41. Would you like to receive training on how to get a good medical photo on smartphones?

- ☐ Yes
- ☐ No
- ☐ Neutral

42. Would you like to receive training in the security, transmission and storage of clinical photographs on smartphones?

- ☐ Yes
- ☐ No
- ☐ Neutral

43. Other comments: (non-obligatory open-ended question)
