# Supplementary material for: Medical Photography in Dermatology: Quality and Safety in the Referral Process to Secondary Healthcare
Source: Diagnostics (Basel). 2025 Jun 14;15(12):1518. doi: 10.3390/diagnostics15121518 (PMC12191595; doi:10.3390/diagnostics15121518)
Supplement: Supplementary file 1 [file diagnostics-15-01518-s001.zip › Supplementary Material S2 Tables.pdf]

Supplementary Material S2

Table - Demographics

Demographics		Total	General and Family Medicine	Dermatology	*p*-value
**Gender**	Male	18 (27.7%)	12 (25.5%)	6 (33.3%)	0.529
	Female	47 (72.3%)	35 (74.5%)	12 (66.6%)	
**Age**	20-40y	39 (60.0%)	29 (61.7%)	10 (55.6%)	0.891
	41-60y	20 (30.8%)	14 (28.8%)	6 (33.3%)	
	>60y	6 (9.2%)	4 (8.5%)	2 (11.1%)	
**Practice Years**	< 5 years	15 (23.1%)	9 (19.1%)	6 (33.3%)	0.411
	5 – 10 years	12 (18.5%)	10 (21.3%)	2 (11.1%)	
	11 – 20 years old	24 (36.9%)	19 (40.4%)	5 (27.8%)	
	More than 20 years	14 (21.5%)	9 (19.1%)	5 (27.8%)	
**Practitioner Level**	Specialist	15 (23.1%)	9 (19.1%)	6 (33.3%)	0.225
	Resident	50 (76.9%)	38 (80.9%)	12 (66.6%)	
**Smartphone exclusively used for work**	No	61 (93.8%)	46 (97.9%)	15 (83.3%)	0.029
	Yes	4 (6.2%)	1 (2.1%)	3 (16.7%)	

Complete answers from Table 2 (Q21 to Q42).

Questions	Total	General and Family Medicine	Dermatology	*p*-value
**Q21 How often do you take pictures of patients?**				
Several times a day	11 (16.9%)	1(2.1%)	10 (55.6%)	
Daily	5 (7.7%)	2 (4.3%)	3 (16.7%)	
Weekly	37 (56.9%)	32 (68.1%)	5 (27.8%)	**<0.001**
Monthly	11 (16.9%)	11 (23.4%)	0 (0.0%)	
Never	1 (1.5%)	1 (2.1%)	0 (0.0%)	
***Q22 What device do you use to take pictures of patients?**				
Dermatoscope	8 (12.3%)	0 (0.0%)	8 (44.4%)	**<0.001**
Personal Smartphone	59 (90.8%)	42 (89.4%)	17 (94.4%)	1.0
Personal Digital Camera	1 (1.5%)	1 (2.1%)	0 (0.0%)	1.0
Exclusive Digital Camera for Clinical Practice	4 (6.2%)	4 (8.5%)	0 (0.0%)	0,569
Patient Smartphone	2 (3.1%)	2 (4.3%)	0 (0.0%)	1.0
***Q23 What are the main reasons for photographic registration?**				
Documenting the patient's clinical evolution	25 (38.5%)	9 (19.1%)	16 (88.9%)	**<0.001**
Request a second opinion	21 (32.3%)	14 (29.8%)	7 (38.9%)	0.558
For educational purposes	12 (18.5%)	0 (0.0%)	12 (66.7%)	**<0.001**
Research and publication	17 (26.2%)	4 (8.5%)	13 (72.2%)	**<0.001**
Registering a Biopsy Site	10 (15.4%)	0 (0.0%)	10 (55.6%)	**<0.001**
Document the appearance of a wound after surgery	15 (23.2%)	3 (6.4%)	12 (66.7%)	**<0.001**
Referral for hospital healthcare	46 (70.8%)	45 (95.7%)	1 (5.6%)	**<0.001**
***Q24 When taking a photo, what characteristics do you consider?**				
Light	43 (66.2%)	28 (59.6%)	15 (83.3%)	0.085
General context	35 (53.8%)	20 (42.6%)	15 (83.3%)	**0.005**
Subject of the photograph in a central position	40 (61.5%)	26 (55.3%)	14 (77.8%)	0.154
Show anatomical marks or scales for later reference	32 (49.2%)	21 (44.7%)	11 (61.1%)	0.277
Photo taken perpendicular to the subject	9 (13.8%)	5 (10.6%)	4 (22.2%)	0.248
Focus	30 (46.2%)	17 (36.2%)	13 (72.2%)	**0.013**
Image sharpness	22 (33.8%)	13 (27.7%)	9 (50.0%)	0.142
True-to-life Colour	55 (84.6%)	39 (83.0%)	16 (88.9%)	0.713
White Balance	4 (22.2%)	0 (0.0%)	4 (6.2%)	**0.005**
Do not include unnecessary elements (e.g. earrings, bracelets, etc.)	14 (21.5%)	6 (12.8%)	10 (44.4%)	**0.015**
**Q25 Do you feel confident in your ability to take an accurate clinical photograph?**				
Yes, I feel confident in my abilities.	22 (33.8%)	13 (27.7%)	9 (50.0%)	
Yes, but there is still room for improvement.	28 (43.1%)	21 (44.7%)	7 (38.9%)	0.268
I feel partially confident	11 (16.9%)	10 (21.3%)	1 (5.6%)	
I don't feel confident when I take a clinical photo.	4 (6.2%)	3 (6.4%)	1 (5.6%)	
**Q26 At some point in your career, did you have training in clinical photography?**				
Yes	4 (93.8%)	0 (0.0%)	4 (22.2%)	**0.005**
No	61 (6.2%)	47 (100.0%)	14 (77.8%)	
**Q27 How often do you ask for consent when taking a photo?**				
I always ask	59 (90.8%)	45 (95.7%)	14 (77.8%)	
I ask often	3 (4.6%)	0 (0.0%)	3 (16.7%)	**0.015**
When I remember or rarely	3 (4.6%)	2 (4.3%)	1 (5.6%)	
***Q28 How do you ask the patient for consent when taking a photo?**				
Verbally	55 (84.6%)	37 (78.7%)	18 (100.0%)	0.051
Written	14 (21.5%)	12 (25.5%)	2 (11.1%)	0.316
Routine recording in the clinical diary	19 (29.2%)	18 (38.3%)	1 (5.6%)	**0.013**

Express consent	4 (6.2%)	4 (8.5%)	0 (0.0%)	0.569
Implied consent	3 (4.6%)	2 (4.3%)	1 (5.6%)	1.0
Patients themselves share images taken by them before the appointment	11 (16.9%)	10 (21.3%)	1 (5.6%)	0.265
***Q29 Where do you store patient photos?**				
Personal Device	28 (43.1%)	11 (23.4%)	17 (94.4%)	**<0.001**
Personal Device for practice only	8 (12.3%)	4 (8.5%)	4 (22.2%)	0.202
Institutional Device	24 (36.9%)	20 (42.6%)	4 (22.2%)	0.159
Clinical patient diary	19 (29.2%)	18 (38.3%)	1 (5.6%)	**0.013**
Specific institutional server	4 (6.2%)	3 (6.4%)	1 (5.6%)	1.0
Personal Cloud (Google Drive, iCloud, Others)	1 (1.5%)	0 (0.0%)	1 (5.6%)	0.277
Institutional hard drive or equivalent	10 (15.4%)	10 (21.3%)	0 (0.0%)	0.051
Never Store	2 (3.1%)	2 (4.3%)	0 (0.0%)	1.0
Not applicable	2 (3.1%)	2 (4.3%)	0 (0.0%)	1.0
**Q30 When do you delete photos from your device?**				
Immediately after storing them elsewhere.	24 (36.9%)	22 (46.8%)	2 (11.1%)	
When I remember after storing them somewhere other than the device.	10 (15.4%)	6 (12.8%)	4 (22.2%)	**0.030**
They always stay on the device.	25 (38.5%)	14 (29.8%)	11 (61.1%)	
It does not apply.	6 (9.2%)	5 (10.6%)	1 (5.6%)	
***Q31 Do you use any of the following methods to protect your information?**				
Access code	17 (26.2%)	8 (17.0%)	9 (50.0%)	**0.011**
Automatic phone lock	20 (30.8%)	9 (19.1%)	11 (61.1%)	**0.002**
Encryption	3 (4.6%)	1 (2.1%)	2 (11.1%)	0.183
Not applicable	35 (53.8%)	31 (66.0%)	4 (22.2%)	**0.002**
***Q32 What method do you use to send or receive clinical photos?**				
Email	37 (56.9%)	30 (63.8%)	7 (38.9%)	0.095
WhatsApp or Facebook Messenger	13 (20.0%)	5 (10.6%)	8 (44.4%)	**0.005**
Application specifically designed for clinical practice.	15 (23.1%)	15 (31.9%)	0 (0.0%)	**0.006**
Not applicable	6 (9.2%)	1 (2.1%)	5 (27.8%)	**0.005**
**Q33 Who do you usually send clinical photographs to?**				
Dermatology	59 (90.8%)	46 (97.9%)	13 (72.2%)	
Other specialties	1 (1.5%)	1 (2.1%)	0 (0.0%)	**<0.001**
Not applicable	5 (7.7%)	0 (0.0%)	5 (27.8%)	
**Q34 How would you rate the quality of the photos you submit in general?**				
Low	5 (7.7%)	4 (8.5%)	1 (5.6%)	
Average	32 (49.2%)	25 (53.2%)	7 (38.9%)	0.292
High or very high	27 (41.5%)	18 (38.3%)	9 (50.0%)	
Not applicable	2 (3.1%)	0 (0.0%)	1 (5.6%)	
**Q35 How important is it for you, as part of effective patient management, to be able to send and receive clinical photos?**				
Very important	20 (30.8%)	11 (23.4%)	9 (50.0%)	
Important	33 (50.8%)	24 (51.1%)	9 (50.0%)	
Kinda important	8 (12.3%)	8 (17.0%)	0 (0.0%)	0.180
Neutral	2 (3.1%)	2 (4.3%)	0 (0.0%)	
Not important	1 (1.5%)	1 (2.1%)	0 (0.0%)	
Not relevant to my clinical practice	1 (1.5%)	1 (2.1%)	0 (0.0%)	
**Q36 Do you consider that you usually contextualize the clinical case of the photograph sent in an appropriate way?**				
Yes, always	24 (36.9%)	18 (38.3%)	6 (33.3%)	
Yes, most of the time	38 (58.5%)	29 (61.7%)	9 (50.0%)	**0.042**
No	1 (1.5%)	0 (0.0%)	1 (5.6%)	
Not applicable	2 (3.1%)	0 (0.0%)	2 (11.1%)	
**Q37 How often do you receive a clinical photo by message or email?**				
Daily	8 (12.3%)	1 (2.1%)	7 (38.9%)	**<0.001**

Weekly	17 (26.2%)	9 (19.1%)	8 (44.4%)	
Monthly	13 (20.0%)	11 (23.4%)	2 (11.1%)	
Rarely	14 (21.5%)	13 (27.7%)	1 (5.6%)	
I don't receive	13 (20.0%)	13 (27.7%)	0 (0.0%)	
***Q38 Who sends you clinical photos?**				
Dermatology	6 (9.2%)	0 (0.0%)	6 (33.3%)	**<0.001**
General and Family Medicine	11 (16.9%)	3 (6.4%)	8 (44.4%)	**<0.001**
Patient	41 (63.1%)	33 (70.2%)	8 (44.4%)	0.084
Other specialties	7 (10.8%)	0 (0.0%)	7 (38.9%)	**<0.001**
Not applicable	15 (23.1%)	13 (27.7%)	2 (11.1%)	0.201
**Q39 Does your workplace have a formal procedure or method for adding clinical photos from your smartphone to a patient's record?**				
Yes	7 (10.8%)	3 (6.4%)	4 (22.2%)	
No	54 (83.1%)	42 (89.4%)	12 (66.7%)	0.089
I'm not sure.	4 (6.2%)	2 (4.3%)	2 (11.1%)	
**Q40 Does your workplace provide clear guidelines on the use of smartphones for clinical photography?**				
Yes	1 (1.5%)	1 (2.1%)	0 (0.0%)	
No	59 (90.8%)	44 (93.6%)	15 (83.3%)	0.208
I’m not sure	5 (7.7%)	2 (4.3%)	3 (16.7%)	
**Q41 Would you like to receive training on how to get a good medical photo on smartphones?**				
Yes	36 (55.4%)	28 (59.6%)	8 (44.4%)	
No	15 (23.1%)	10 (21.3%)	5 (27.8%)	0.542
Neutral	14 (21.5%)	9 (19.1%)	5 (27.8%)	
**Q42 Would you like to receive training in the security, transmission and storage of clinical photographs on smartphones?**				
Yes	43 (66.2%)	30 (63.8%)	13 (72.2%)	
No	12 (18.5%)	11 (23.4%)	1 (5.6%)	0.208
Neutral	10 (15.4%)	6 (12.8%)	4 (22.2%)	

Questions marked with * have multiple answers
